# Atorvastatin 10 mg plus ezetimibe versus titration to atorvastatin 40 mg: attainment of European and Canadian guideline lipid targets in high-risk subjects ≥65 years

**DOI:** 10.1186/1476-511X-13-13

**Published:** 2014-01-13

**Authors:** Christian Constance, Ori Ben-Yehuda, Nanette K Wenger, Franklin Zieve, Jianxin Lin, Mary E Hanson, Robert S Lowe, Andrew M Tershakovec

**Affiliations:** 1 Hopital Maisonneuve-Rosemont Recherche Clinique/Polyclinique, 5415 Boulevard de l’Assomption, Suite 295, H1T 2 M4 Montreal, QC, Canada; 2 Clinical Trial Center, Cardiovascular Research Foundation, New York, NY, USA; 3 Emory University School of Medicine, Altanta, GA, USA; 4 McGuire VA Medical Center, Richmond, VA, USA; 5 Merck & Co. Inc., Whitehouse Station, NJ, USA

**Keywords:** Ezetimibe, Atorvastatin, Hyperlipidemia, Elderly, Statin, Combination therapy

## Abstract

**Background:**

Few clinical studies have focused on the efficacy of lipid-lowering therapies in patients ≥65 years.

**Methods:**

After stabilization on atorvastatin 10 mg, hypercholesterolemic subjects ≥65 years at high/very high risk for CHD and not at LDL-C <1.81 mmol/L (with atherosclerotic vascular disease [AVD]) or <2.59 mmol/L (without AVD) were randomized to ezetimibe 10 mg plus atorvastatin 10 mg or uptitration to atorvastatin 20 mg (6 weeks) followed by uptitration to 40 mg (additional 6 weeks). A *post-hoc* analysis compared between-group differences in percent attainment of individual and combined LDL-C, non-HDL-C and Apo B targets based on recommendations from 2012 European and Canadian Cardiovascular Society (CCS) guidelines for dyslipidemia treatment.

**Results:**

Atorvastatin 10 mg plus ezetimibe produced significantly greater attainment of LDL-C, non-HDL-C, and Apo B individual and dual/triple targets vs. atorvastatin 20 mg for the entire cohort and very high-risk groups at 6 weeks. After 12 weeks, very high-risk subjects maintained significantly greater achievement of LDL-C <1.8 mmol/L (47% vs. 35%), non-HDL-C <2.6 mmol/L (63% vs. 53%) and Apo B <0.8 g/L (47% vs. 38%) single targets and dual/triple targets with atorvastatin 10 mg plus ezetimibe vs. atorvastatin 40 mg, while attainment of European target for high-risk subjects was generally similar for both treatments. Achievement of Canadian targets was significantly greater with combination therapy vs. atorvastatin 20 mg (6 weeks) or atorvastatin 40 mg (12 weeks).

**Conclusions:**

Atorvastatin 10 mg plus ezetimibe provided more effective treatment than uptitration to atorvastatin 20/40 mg for attainment of most European and Canadian guideline-recommended lipid targets in older at-risk patients.

**Trial registration:**

ClinicalTrials.gov identifier NCT00418834.

## Introduction

Cardiovascular disease (CVD) is a leading contributor to global morbidity and mortality, accounting for 40% of all deaths in the European Union and about one-third of all deaths in Canada (~29%) [1,2]. Canadian and European guidelines have identified low-density lipoprotein cholesterol (LDL-C) as the primary therapeutic target for treatment of patients with hypercholesterolemia and CVD or CVD risk factors [3,4]. The Canadian guidelines and the European guidelines recommend apolipoprotein (Apo) B, and non-high-density lipoprotein (HDL)-C as secondary targets and total cholesterol is recommended as a secondary target by the European guidelines [3,4]. While the Canadian guidelines specify that treatment targets should not change based on risk assessment, the European guidelines designate different levels based on risk; more aggressive goals are recommended for patients at very high risk for CVD relative to those at high or moderate CVD risk [4]. Although increasing age is a risk factor for CVD, neither set of guidelines recommends specific targets based on age. Many patients require aggressive LDL-C lowering strategies to achieve some of the more aggressive lipid-lowering targets [5-8]. In addition, there is strong justification for intensive LDL-C-lowering therapy in individuals older than 65 years with established CVD [7].

Although aggressive therapy with high-dose statins has been shown to provide clinical benefit [5], it is not always an option in elderly patients. Concerns about tolerability exist due to differences in metabolism, greater likelihood of polypharmacy, and potentially greater risk of myopathy. Combination therapy, such as the addition of ezetimibe 10 mg to atorvastatin 10 mg, may deliver complementary effects on lipids that surpass those of atorvastatin monotherapy titrated to higher doses, while providing comparable tolerability. This post hoc analysis of data from the ZETia in the ELDerly (ZETELD) study [9] assessed the effects of adding ezetimibe to atorvastatin 10 mg versus doubling the atorvastatin dose to 20 mg (first 6 weeks) followed by uptitration to atorvastatin 40 mg for an additional 6 weeks on the attainment of specified lipid targets in patients ≥65 years with hypercholesterolemia, based on recommendations for targets defined by the 2012 European and 2012 Canadian guidelines for treatment of dyslipidemias [3,4].

## Methods

### Trial design

This *post hoc* analysis evaluated data from the previously reported 12-week multicenter, randomized, double-blind, parallel-arm ZETELD study (ClinicalTrials.gov identifier NCT00418834; protocol number 112) [9]. The investigational review boards of each participating study site reviewed and approved the protocol and amendments, and all subjects provided written informed consent prior to performance of any study procedure.

### Subjects and therapy

Details of the study have been previously described [9]. Briefly, participants eligible for inclusion were males and females ≥65 years with or without atherosclerotic vascular disease (AVD) who had not reached LDL-C target levels of <1.81 mmol/L (those with AVD) or <2.59 mmol/L (those without AVD) during atorvastatin 10 mg/d therapy.

Subjects were eligible for study inclusion if they met the following criteria: established coronary heart disease (CHD) and other AVD with LDL-C levels ≥1.81 mmol/L and ≤4.14 mmol/L; subjects without AVD who had diabetes mellitus (type 1 or 2), or multiple risk factors and a 10-year risk for CHD >20% (as determined by the Framingham calculation) [10,11] and LDL-C levels ≥2.59 mmol/L and ≤4.92 mmol/L; triglycerides ≤3.96 mmol/L; alanine aminotransferase and aspartate aminotransferase ≤1.5 times the upper limit of normal (ULN) with no active liver disease; creatine kinase ≤2 times the ULN; thyroid stimulating hormone ≥0.3 or ≤5.0 μIU/mL; and HbA1c <8.5%. Patients were excluded from the study if they had uncontrolled hypertension (systolic blood pressure >160 mmHg or diastolic >100 mmHg), impaired renal function (creatinine ≥2.0 mg/d or a history of nephrotic range proteinuria), or were taking a lipid-lowering agent with greater potency than atorvastatin 20 mg (acceptable treatment included atorvastatin 10-20 mg, simvastatin 10-40 mg, pravastatin 10-40 mg, fluvastatin 20-80 mg, lovastatin 10-40 mg, rosuvastatin 5 mg, or ezetimibe 10 mg) within 6 weeks or fibrates within 8 weeks of screening. Subjects enrolled in the study received single-blind atorvastatin 10 mg daily during a 4 week run-in period. Following the run-in period, qualified patients were randomized to receive ezetimibe and atorvastatin or atorvastatin alone. Randomization was stratified based on baseline LDL-C levels and the presence or absence of AVD to achieve balance across treatment groups (1: LDL-C ≥70 to <100 mg/dL with AVD; 2: LDL-C ≥100 to <130 mg/dL with AVD; 3: LDL-C ≥100 to <130 mg/dL without AVD; 4: LDL-C ≥130 to ≤160 mg/dL with AVD; 5: LDL-C ≥130 to <160 without AVD; 6: LDL-C ≥160 to ≤190 without AVD [LDL-C conversions: 70 mg/dL = 1.81 mmol/L, 100 mg/dL = 2.59 mmol/L, 130 mg/dL = 3.37 mmol/L, 160 mg/dL = 4.14 mmol/L, 190 mg/dL = 4.92 mmol/L]). During the first 6 weeks, patients received ezetimibe 10 mg plus atorvastatin 10 mg daily, or atorvastatin 20 mg daily. After the first 6 weeks, subjects in the ezetimibe 10 mg plus atorvastatin 10 mg group continued on the same treatment for an additional 6 week period while subjects in the atorvastatin 20 mg group were titrated to atorvastatin 40 mg for an additional 6 weeks.

### Statistical methods

Consistent with the main study, this *post hoc* analysis included all randomized subjects with a baseline and at least one post-baseline evaluation. The proportion of subjects achieving risk-based LDL-C, non-HDL-C, and Apo B treatment targets as defined in the current European guidelines on cardiovascular disease prevention in clinical practice (version 2012) [4] and the 2012 Canadian Cardiovascular Society/Canadian guidelines for treatment of dyslipidemia [3], were assessed after 6 weeks and after 12 weeks of treatment by logistical regression with a term for treatment. Attainment of individual targets, dual targets of LDL-C and either non-HDL-C or Apo B, as well as the combination of all three targets was evaluated for all randomized subjects and for subgroups based on risk when evaluating targets defined in the European guidelines. Statistical significance for between-treatment differences was determined using 95% confidence intervals for odds ratios which excluded 1. This was a *post hoc* analysis. No adjustments for multiplicity were made, thus the overall type 1 error rate is greater than 0.05. Target levels based on Canadian guidelines included LDL-C ≤2.0 mmol/L, non-HDL-C ≤2.6 mmol/L and Apo B ≤0.8 g/L. Target levels based on European guidelines included LDL-C <2.5 mmol/L, non-HDL-C <3.3 mmol/L, and Apo B <1.0 g/L for subjects at high risk for (i.e. those without AVD) and LDL-C <1.8 mmol/L, non-HDL-C <2.6 mmol/L, and Apo B <0.8 g/L for subjects at very high risk (i.e. those with AVD). Of note, dual and triple targets are not explicitly stated in either the European or Canadian guidelines, but were defined *a priori* for these analyses. Safety and tolerability were evaluated previously [9,12] and were not included in this *post hoc* analysis.

## Results

Of 2276 subjects screened for this study, 1053 were randomized; 526 to atorvastatin 10 mg plus ezetimibe 10 mg add-on, and 527 to atorvastatin 20/40 mg. Patient discontinuation after randomization (23 receiving atorvastatin plus ezetimibe, 20 receiving atorvastatin 20/40), baseline demographics (including the National Cholesterol Education Program, Adult Treatment Panel III [NCEP ATP III] defined risk), and baseline lipid values were similar between treatment groups and previously described [9]. Overall, the mean age was 71 years (±5), 53% were women, 96% were white, approximately 30% were obese (body mass index ≥30 kg/m^2^), ≥80% had CHD or AVD, and the mean treated baseline LDL-C levels ranged from 2.62 and 2.67 mmol/L. Baseline lipid levels along with recommended lipid targets from the European and Canadian guidelines are shown in Table 1. Baseline lipids were generally similar between treatment groups and subgroups by AVD status.

**Table 1:** Current European and Canadian guideline recommendations for lipids, lipoproteins, and baseline levels for study participants

**Parameter**		**Subjects without AVD**				**Subjects with AVD**			
		**Guidelines**		**Treatment group**		**Guidelines**		**Treatment group**	
		CCS^*^	**EU**^**†**^**high risk**	**E + A10**	**A20**	CCS^*^	**EU**^**†**^** very high risk**	**E + A10**	**A20**
				**n = 66**^**‡**^	**n = 67**^**‡**^			**n = 449**^**‡**^	**n = 448**^**‡**^
LDL-C	(mmol/L)	≤2.0 or	<2.5	3.25 (0.55)	3.11 (0.53)	≤2.0 or	<1.8	2.58 (0.70)	2.56 (0.49)
		↓50%				↓50%	or ↓50%		
Non-HDL-C	(mmol/L)	≤2.6	<3.3	4.04 (0.64)	3.84 (0.65)	≤2.6	<2.6	3.21 (0.76)	3.21 (0.61)
Apo B	(g/L)	≤0.8	<1.0	1.2 (0.2)	1.1 (0.2)	≤0.8	<0.8	1.0 (0.2)	1.0 (0.2)

Baseline lipid and lipoprotein levels were determined for parameters common to CCS2012 and EU2012 targets.

A 10/20, atorvastatin 10 or 20 mg; Apo, apolipoprotein; AVD, atherosclerotic vascular disease; CCS, Canadian Cardiovascular Society guidelines (2012); E, ezetimibe 10 mg; EU, European Guidelines (2012); LDL-C, low-density lipoprotein cholesterol; non-HDL-C, non-high-density lipoprotein cholesterol.

*Canadian guidelines specify that treatment targets should not change based on risk assessment.

^†^European guidelines state that total cholesterol should be considered a treatment target if other analyses are not available.

^‡^n may vary slightly between measured parameters due to differences in available data.

### Efficacy endpoints

**Figure 1:**
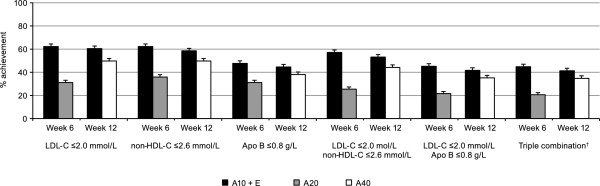
Proportion of subjects achieving targets defined by Canadian guidelines*. Error bars = standard error. *Canadian Cardiovascular Society/Canadian Dyslipidemia guidelines (2012) [4]. † Triple combination includes LDL-C ≤2.0 mmol/L, non-HDL-C ≤2.6 mmol/L, and Apo B ≤0.8 g/L. A, atorvastatin (10, 20 or 40 mg); Apo, apolipoprotein; AVD, atherosclerotic vascular disease; E, ezetimibe 10 mg; LDL-C, low-density lipoprotein cholesterol; non-HDL-C, non-high-density lipoprotein cholesterol.

The proportion of subjects achieving targets defined by the 2012 Canadian guidelines for LDL-C, non-HDL-C, and Apo B are shown in Figure 1. At 6 weeks, the percentage of subjects with LDL-C ≤2.0 mmol/L was 62.3% with ezetimibe add-on to atorvastatin 10 mg compared with 31.1% with atorvastatin titration to 20 mg. A greater proportion of subjects also attained the single targets of non-HDL-C ≤2.6 mmol/L and Apo B ≤0.8 g/L, as well as dual and triple combination targets with ezetimibe plus atorvastatin combination therapy. The odds ratios were significant for all comparisons (Table 2). After an additional 6 weeks, subjects continuing on ezetimibe plus atorvastatin 10 mg maintained a similar level of attainment for single, dual, and triple targets, while percent attainment of targets for subjects titrated from atorvastatin 20 mg to atorvastatin 40 mg showed an approximately 19 percentage point increase in LDL-C target attainment, 14 percentage point increase in non-HDL-C target attainment, 7 percentage point increase in Apo B target attainment, and a 14-19 percentage point increase in dual/triple target attainment. The proportion of subjects reaching single, dual, or triple targets at 12 weeks was greater for ezetimibe add-on therapy (Table 2).

**Table 2:** Percent achievement and odds ratios for LDL-C, non-HDL-C and Apo B targets

	**All subjects**						**High risk or very high risk subjects**					
	**Week 6**			**Week 12**			**Week 6**			**Week 12**		
**Targets**	**E + A10**	**A20**	**OR**	**E + A10**	**A20/A40**	**OR**	**E + A10**	**A20**	**OR**	**E + A10**	**A20/A40**	**OR**
(mmol/L) for LDL-C, non-HDL-C; g/L for Apo B												
**Canadian**	(N = 515)	(N = 515)		(N = 516)	(N = 509)							
Single												
LDL-C ≤2.0	62.3	31.1	3.67^*^	60.5	49.7	1.55^*^						
non-HDL-C ≤2.6	62.3	35.9	2.95^*^	58.5	49.7	1.43^*^						
Apo B ≤0.8	47.7	31.1	2.02^*^	44.6	38.1	1.31^*^						
Dual/Triple												
LDL-C ≤2.0, non-HDL-C ≤2.6	57.1	25.4	3.90^*^	53.1	44.2	1.43^*^						
LDL-C ≤2.0, Apo B ≤1.0	45.2	21.6	2.99^*^	41.7	35.2	1.32^*^						
LDL-C ≤2.0, non-HDL ≤2.6, Apo B ≤0.8	44.8	20.6	3.12^*^	41.3	34.8	1.32^*^						
**EU-High Risk**	(N = 515)	(N = 515)		(N = 516)	(N = 516)		(n = 66)	(n = 67)		(n = 67)	(n = 67)	
Single												
LDL-C <2.5	85.2	72.6	2.18^*^	82.4	77.0	1.39^*^	68.2	50.7	2.08^*^	61.2	61.2	**1.00**
non-HDL-C <3.3	86.2	78.1	1.76^*^	84.1	81.9	**1.17**	68.2	59.7	**1.45**	68.7	70.1	**0.93**
Apo B <1.0	78.3	69.5	1.59^*^	74.8	71.5	**1.18**	58.7	48.5	**1.51**	53.8	53.0	**1.03**
Dual/Triple												
LDL-C <2.5, non-HDL-C <3.3	83.9	70.3	2.20^*^	81.0	74.3	1.48^*^	66.7	50.7	**1.94**	61.2	58.2	**1.13**
LDL-C <2.5, Apo B <1.0	76.3	63.6	1.84^*^	72.8	67.8	**1.27**	55.6	40.9	**1.81**	49.2	50.0	**0.97**
LDL-C <2.5, non-HDL <3.3, Apo B <1.0	75.9	63.0	1.85^*^	72.6	67.2	**1.29**	55.6	40.9	**1.81**	49.2	50.0	**0.97**
**EU-Very High Risk**	(N = 515)	(N = 515)		(N = 516)	(N = 516)		(n = 449)	(n = 448)		(n = 449)	(n = 442)	
Single												
LDL-C <1.8	47.4	17.9	4.14^*^	43.6	32.2	1.63^*^	50.8	20.1	4.10^*^	47.2	35.3	1.64^*^
Non-HDL-C <2.6	62.3	35.9	2.95^*^	58.5	49.7	1.43^*^	66.1	39.7	2.96^*^	62.6	52.5	1.51^*^
Apo B <0.8	45.4	28.0	2.13^*^	43.8	35.4	1.43^*^	48.9	30.8	2.15^*^	46.9	37.7	1.46^*^
Dual/Triple												
LDL-C <1.8, non-HDL-C <2.6	45.4	15.1	46.7^*^	41.3	30.1	1.64^*^	48.8	17.4	4.52^*^	45.0	32.8	1.68^*^
LDL-C <1.8, Apo B <0.8	38.7	14.4	3.75^*^	35.2	25.9	1.56^*^	41.4	16.3	3.64^*^	38.1	28.2	1.57^*^
LDL-C <1.8, non-HDL <2.6, Apo B <0.8	38.3	13.6	3.93^*^	35.2	25.7	1.57^*^	41.0	15.6	3.75^*^	38.1	28.0	1.59^*^

N may vary slightly between measured parameters due to differences in available data.

*OR considered significant based on associated 95% confidence interval which excludes 1.

A 10/20, atorvastatin 10 or 20 mg; Apo, apolipoprotein; AVD, atherosclerotic vascular disease; Canadian, Canadian Cardiovascular Society guidelines (2012); E, ezetimibe 10 mg; EU, European Guidelines (2012); LDL-C, low-density lipoprotein cholesterol; non-HDL-C, non-high-density lipoprotein cholesterol; OR, odds ratio.

**Figure 2:**
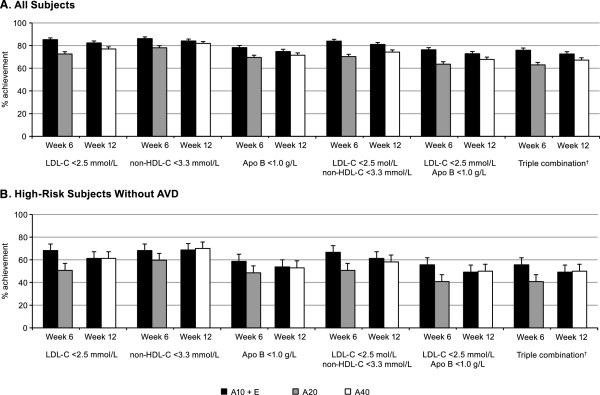
Proportion of all and high-risk subjects achieving targets defined by European guidelines*. A. All Subjects. **B**. High-Risk Subjects without AVD. Error bars = standard error. * European Guidelines on cardiovascular disease prevention in clinical practice (version 2012) [12]. † Triple combination includes LDL-C <2.5 mmol/L, non-HDL-C <3.3 mmol/L, and Apo B <1.0 g/L. A, atorvastatin (10, 20 or 40 mg); Apo, apolipoprotein; AVD, atherosclerotic vascular disease; E, ezetimibe 10 mg; LDL-C, low-density lipoprotein cholesterol; non-HDL-C, non-high-density lipoprotein cholesterol.

Target levels for LDL-C <2.5 mmol/L, non-HDL-C <3.3 mmol/L, and Apo B <1.0 g/L for subjects at high risk were evaluated based on recommendations from the European Guidelines as single, dual, and triple target combinations for the entire patient population as well as for subjects at high risk without AVD (Figure 2). When all subjects were included in the analysis, after 6 weeks of therapy attainment of single, dual, and triple target combinations was achieved by a greater proportion of subjects receiving ezetimibe add-on to atorvastatin 10 mg compared with subjects titrated to atorvastatin 20 mg (Table 2). At 12 weeks, attainment was numerically higher with ezetimibe plus atorvastatin compared with up titration to atorvastatin 40 mg for all targets, but the odds ratios were only significant for LDL-C <2.5 mmol/L alone or for the dual target of LDL-C <2.5 mmol/L and non-HDL-C <3.3 mmol/L (Table 2). For high risk subjects without AVD, attainment of single, dual, and triple targets after 6 weeks of treatment were numerically greater with ezetimibe plus atorvastatin 10 mg than atorvastatin 20 mg, with predictive odds that were significant only for LDL-C <2.5 mg/dL. After 12 weeks, the proportion of subjects achieving each of the target levels was similar between treatments. The level of target attainment by subjects at high risk without AVD was approximately 15-20 percentage points lower than that for the entire patient population.

**Figure 3:**
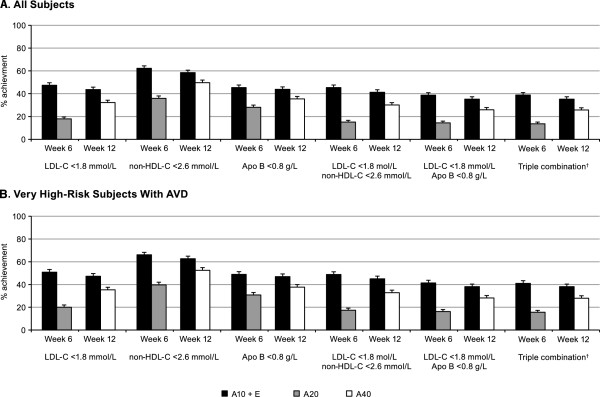
Proportion of all and very high-risk subjects achieving targets defined by European guidelines*. A. All Subjects. **B**. Very High-Risk Subjects with AVD. Error bars = standard error. * European Guidelines on cardiovascular disease prevention in clinical practice (version 2012) [12]. † Triple combination includes LDL-C <1.8 mmol/L, non-HDL-C <2.6 mmol/L, and Apo B <0.8 g/L. A, atorvastatin (10, 20 or 40 mg); Apo, apolipoprotein; AVD, atherosclerotic vascular disease; E, ezetimibe 10 mg; LDL-C, low-density lipoprotein cholesterol; non-HDL-C, non-high-density lipoprotein cholesterol.

European guideline targets defined for subjects with very high risk include LDL-C <1.8 mmol/L, non-HDL-C <2.6 mmol/L, and Apo B <0.8 g/L; achievement of these levels as single, dual, and triple target combinations were evaluated for all subjects and for subjects at very high risk with AVD (Figure 3). After 6 weeks of treatment for the entire study cohort, achievement of all target levels was approximately 1.5 to 3 times higher with ezetimibe add-on to atorvastatin 10 mg than with atorvastatin 20 mg. These differences were statistically significant (Table 2). At 12 weeks, the level of attainment was maintained for ezetimibe plus atorvastatin and increased by 7-15 percentage points after titration to atorvastatin 40 mg, with all odds ratios significantly in favor of combined therapy. Attainment levels of single, dual, and triple targets for subjects at very high risk with AVD were similar to that observed for the full study cohort, with all odds ratios being significant after 6 weeks (range from 2.15 to 4.52) and 12 weeks (range from 1.46 to 1.68) of therapy.

## Discussion

Epidemiological studies have identified several risk factors for CVD. These include both non-modifiable factors such as age, family history, and male sex; and modifiable factors, such as smoking, blood pressure, and cholesterol levels. The identification and management of these modifiable risk factors is an important step for reducing the risk of CVD and cardiovascular events in patients at any age.

Treatment with more intensive statin doses has been shown to have significantly greater effects than moderate statin therapy on cholesterol reduction in the general population [13]; and the use of intensive lipid-altering therapy is supported in older subjects as well [5,6]. A recent observational study of clinical practice in Europe and Canada highlights the need for better management of dyslipidemia, with almost half of the 22,063 statin-treated patients studied (mean age of 65.7 ± 9.9 years) having LDL-C levels above guideline recommended targets [13].

The present *post hoc* analysis showed that in subjects aged 65 years and older, the odds of achieving the LDL-C, non-HDL-C and Apo B treatment targets recommended by the 2012 Canadian and 2012 European treatment guidelines were greater with the combination of atorvastatin 10 mg plus ezetimibe 10 mg compared with uptitration of atorvastatin to 20 mg for 6 weeks and 40 mg for an additional 6 weeks. Moreover, in the subgroup of subjects at very high risk, the odds of achieving the most aggressive lipid targets were greater with the combination compared with uptitration at both 6 weeks and 12 weeks, similar to the odds of target achievement in the overall population. In the subgroup of subjects at high risk; however, the odds of achieving most of the recommended targets were similar for both treatments at both 6 weeks and 12 weeks. These results were consistent with the primary analysis which evaluated the attainment of LDL-C <70 mg/dL or LDL-C <100 or <70 mg/dL for all subjects.

It has been suggested that statin therapy should be used more conservatively in older patients, especially in those with an increased susceptibility to adverse events due to concomitant illnesses and/or medications [14]. Although the Study Assessing Goals in the Elderly (SAGE) demonstrated greater benefits of intensive versus moderate statin therapy, suggesting that the benefits may outweigh the concerns, caution is advised when prescribing statins in individuals older than 65 years due to increased risk of myopathy. The United States Food and Drug Administration has restricted the use of the 80 mg dose of simvastatin in populations at any age [15-17]. Combination therapy with drugs that have different mechanisms of action may be beneficial for lipid-lowering in older patients. A pooled analysis that included 16 studies showed that ezetimibe used in combination with a statin lowered LDL-C 17% more than statin monotherapy at equivalent doses in those aged under 65, 65-74 and 75 years and older [18]. In addition, the proportion of subjects achieving LDL-C <100 mg/dL or <70 mg/dL was greater with the combination therapy versus statin monotherapy in all age groups [18]. The current analyses are consistent with these previous reports, suggesting that therapy with ezetimibe added to moderate doses of atorvastatin may provide a valuable lipid-lowering option for older men and women at high and very high risk for CVD. Of note, whether LDL-C reductions attributed to combination treatment versus statin monotherapy will result in better CVD outcomes is not yet known and awaits results from an ongoing clinical trial.

### Study limitations

Compared with the overall population and the very high risk subgroup, which had similar group sizes to each other, the high risk subgroup in this analysis was relatively small and underpowered to show statistical significance based on associated 95% confidence intervals. While odds ratios for the high risk subjects were generally consistent with the odds ratios for the overall population, limited sample size prohibits making definitive statements concerning the significance of these differences. The results of these analyses were based on a study of relatively short duration in a population that was predominantly white (96%), which limits the ability to generalize the conclusions to longer-term therapy and diverse populations. Of note, the guidelines also include a 50% LDL-C reduction; but we could not assess this as we did not have baseline naïve LDL-C levels. Finally, these were *post hoc* analyses; no inferential statistics were provided and no adjustments for multiplicity were made. Therefore, although the results provide information about the efficacy of ezetimibe combined with atorvastatin in older subjects, they should be interpreted with discretion.

## Conclusions

These results taken together with the primary findings of the ZETELD study suggest that ezetimibe 10 mg combined with atorvastatin 10 mg may provide an effective treatment option for the management of dyslipidemia and attainment of European and Canadian guideline-recommended targets in patients ages 65 years and older at high and very high risk, with comparable tolerability to higher dose atorvastatin.

## Abbreviations

Apo: Apolipoprotein; AVD: Atherosclerotic vascular disease; CVD: Cardiovascular disease; CHD: Coronary heart disease; HDL-C: High-density lipoprotein cholesterol; LDL-C: Low-density lipoprotein cholesterol; NCEP ATP III: National Cholesterol Education Program, Adult treatment panel III; SAGE: Study assessing goals in the elderly; ULN: Upper limit of normal.

## Competing interests

CC reports receiving personal fees from lectures not related to the submitted work. OBY and FZ report no conflicts of interest. NKW reports grants from Abbott, Amgen, AstraZeneca, Gilead Sciences, Janssen Pharmaceuticals, Merck, NHLBI, and Pfizer, and work with the Abbott Women’s Advisory Board. JL, MEH, RSL and AMT are employees of Merck & Co., Inc. and may own stock or hold stock options in the company.

## Authors’ contributions

Each of the authors fulfilled the ICMJE authorship criteria. They all contributed to the design of the study, collected or assembled the data, performed or supervised analyses, and/or interpreted the results; wrote sections of the initial draft and/or provided substantive suggestions for revisions on subsequent iterations of the manuscript. In addition, all authors had access to study data and approved the final version of this article.
